# Borreliella burgdorferi Antimicrobial-Tolerant Persistence in Lyme Disease and Posttreatment Lyme Disease Syndromes

**DOI:** 10.1128/mbio.03440-21

**Published:** 2022-04-25

**Authors:** Felipe C. Cabello, Monica E. Embers, Stuart A. Newman, Henry P. Godfrey

**Affiliations:** a Department of Pathology, Microbiology and Immunology, New York Medical College, Valhalla, New York, USA; b Department of Cell Biology and Anatomy, New York Medical College, Valhalla, New York, USA; c Division of Immunology, Tulane National Primate Research Center, Tulane University Health Sciences, Covington, Louisiana, USA; McGovern Medical School; University of Texas Health Science Center at Houston

**Keywords:** bacterial persistence, *Borrelia burgdorferi*, Lyme disease, post-Lyme disease syndromes, antimicrobial tolerance, persistence, post-treatment syndromes

## Abstract

The annual incidence of Lyme disease, caused by tick-transmitted Borreliella burgdorferi, is estimated to be at least 476,000 cases in the United States and many more worldwide. Ten to 20% of antimicrobial-treated Lyme disease patients display posttreatment Lyme disease syndrome (PTLDS), a clinical complication whose etiology and pathogenesis remain uncertain. Autoimmunity, cross-reactivity, molecular mimicry, coinfections, and borrelial tolerance to antimicrobials/persistence have been hypothesized and studied as potential causes of PTLDS. Studies of borrelial tolerance/persistence *in vitro* in response to antimicrobials and experimental studies in mice and nonhuman primates, taken together with clinical reports, have revealed that B. burgdorferi becomes tolerant to antimicrobials and may sometimes persist in animals and humans after the currently recommended antimicrobial treatment. Moreover, B. burgdorferi is pleomorphic and can generate viable-but-nonculturable bacteria, states also involved in antimicrobial tolerance. The multiple regulatory pathways and structural genes involved in mediating this tolerance to antimicrobials and environmental stressors by persistence might include the stringent (*rel* and *dksA*) and host adaptation (*rpoS*) responses, sugar metabolism (*glpD*), and polypeptide transporters (*opp*). Application of this recently reported knowledge to clinical studies can be expected to clarify the potential role of bacterial antibacterial tolerance/persistence in Lyme disease and PTLDS.

## INTRODUCTION

There are currently approximately 476,000 new cases in the United States each year of Lyme disease, a tick-borne disease caused by Borreliella burgdorferi (1), and counties now considered to have a high incidence of this disease have recently increased 250 to 300% in the north-central and northeastern states (1–5). The Tick-Borne Disease Working Group, convened by the United States Department of Health and Human Services under the umbrella of the 21^st^ Century Cures Act, has submitted two reports to Congress. The 2018 report noted that “while most Lyme disease patients who are diagnosed and treated early can fully recover, 10% to 20% of patients suffer from persistent symptoms, which for some are chronic and disabling,” a clinical entity designated posttreatment Lyme disease syndrome (PTLDS) (5–8). The report estimated the care of patients with Lyme disease (including PTLDS) to cost approximately $1.3 billion per year. The 2020 Pathogenesis and Pathophysiology of Lyme Disease Subcommittee report recommended prioritizing research in several areas, including “support of targeted funding of research that aims to determine the potential roles of antimicrobial tolerance and immunomodulation in the persistence of B. burgdorferi despite antimicrobial treatment” (9).

It is likely that there are multiple causes of PTLDS. Undertreatment is unlikely, given the results of multiple clinical trials employing retreatment with higher doses of the initial antimicrobial or with different antimicrobials, despite doubts regarding the lack of effect of retreatment (10–13). Possible explanations for PTLDS based on research *in vitro* (14–16) and in animals (17–20) and humans (21) have included coinfections with still-undetected pathogens (5, 9), persistence of antimicrobial-tolerant living and dead B. burgdorferi organisms and their components (14–16, 19–23), and dysfunctional patient immune responses resulting from cross-reactivity/mimicry of bacterial antigens and host tissues triggered by the initial infection or by persistent organisms (5, 9, 24). These mechanisms can clearly interact. Bacterial persistence can result from antigenic changes in the organism, colonization of immunologically protected sites and subversion of the immune response, growth in biofilms, antimicrobial tolerance/persistence and/or exposure to host immune responses (2, 24–28). Autoimmunity can be triggered not only by the initial infection but also in response to borrelial persistence secondary to antimicrobial tolerance. The variation in signs and symptoms of PTLDS in different patient subsets is consistent with such complex immune-related interactions (5, 9, 24).

Recent research on Lyme disease and the biology of B. burgdorferi has occurred in the context of a broader public health crisis of antimicrobial resistance and the discovery of the ability of bacteria to become phenotypically tolerant to antimicrobials and host defenses (26, 27, 29–31). Infected animals can harbor phenotypically antimicrobial-tolerant and viable-but-nonculturable (VBNC) B. burgdorferi organisms capable of producing pathological alterations in the host (20, 32). There is also preliminary evidence that infected patients can harbor such populations (21). Direct correlation of these findings to the pathogenesis of PTLDS remains unexplored, and an integrated approach is needed to obtain a better understanding of the progression of Lyme disease in humans and the potential role of borrelial antimicrobial tolerance/persistence in this progression (5, 9, 12, 21, 24). The goal of this review is therefore to critically examine the extant literature in these disparate areas with the aim of evaluating the potential role and relevance of borrelial antimicrobial tolerance/persistence and its ability to remain in host tissues for extended periods of time (i.e., to persist there) to the pathogenesis of Lyme disease and Lyme arthritis, neuroborreliosis, and PTLDS.

## BACTERIAL ANTIMICROBIAL TOLERANCE/PERSISTENCE

Phenotypic antimicrobial tolerance by persistence was originally identified and defined as the ability of a small fraction of an isogenic bacterial population to escape the antimicrobial activity of a particular agent in the absence of any increase in the agent’s minimum inhibitory concentration for this population (33–35). Antimicrobial-tolerant persistent bacteria display an increase in the minimum time needed to kill 99.99% of the population (MDK_99.99_) as well as heterogeneity in cellular susceptibility in a culture (26, 27, 29, 36–38). These changes, relevant only to bactericidal antibiotics, result in a biphasic mortality curve (26, 27, 36–39). This phenomenon has traditionally been called persistence, and bacteria displaying this phenotype, persisters (26, 27, 29, 36, 37). The term “antimicrobial tolerance/persistence” is used here to describe the small fraction of single cell heterogeneous antimicrobial-tolerant persister cells (37, 38, 40). This avoids conflating the cellular persistence with which we are concerned with the prolonged presence (also called persistence) of B. burgdorferi in its hosts during its life cycle (2, 26, 27, 29, 36–38, 40). Antimicrobial tolerance may also be the result of the whole bacterial culture becoming tolerant to antimicrobials (antimicrobial tolerance *per se*) (38, 40). Distinguishing between these two forms of antimicrobial tolerance is probably critical for a mechanistic understanding of infection relapse: persister cells appear to be more likely than antimicrobial-tolerant cells *per se* to be involved in initiating this process (40).

The antimicrobial-tolerant persister phenotype is an epigenetic rather than a genotypic property (29, 36, 38, 39): reculture of isolated single persister bacteria in fresh medium lacking antimicrobial generates a newly heterogenous bacterial population containing mainly susceptible cells and a small fraction of antimicrobial-tolerant cells with a new biphasic killing curve on re-exposure (36–39). Although the antimicrobial-tolerant phenotype is epigenetic, it can result from genetic mechanisms mediating this tolerance as well as from epigenetic changes, such as DNA methylation, that may display memory effects (26, 27, 37, 39).

Antimicrobial-tolerant persister bacteria and putative VBNC bacteria represent a continuum of antimicrobial tolerance (36–38, 41–43). Depending on the particular bacterial species being studied, this can involve multiple and complex mechanisms (26, 27, 29, 35, 37) including the stringent and SOS responses (26, 27, 29–31, 36, 41, 44, 45), toxin-antitoxin modules (37, 46, 47), protein aggregates (including ribosomes and chaperones) (43, 48–50), quorum sensing (51, 52), efflux pumps (53), decreases in ATP levels (54), and modulations in glycerol metabolism (55). Although antimicrobial-tolerant persistent bacteria can spontaneously emerge during culture, their appearance can also be triggered by nutritional, osmotic, and acidic environmental stresses, growth phase, antimicrobials, and bacterial metabolites, such as quorum sensing mediators (26, 27, 36–39, 44, 52). The mechanisms by which antimicrobial-tolerant bacteria enter and leave the persistent state are poorly understood (26, 27, 30, 31, 43). They may involve stochastic responses to unfavorable or favorable environmental conditions activated by extracellular factors, such as loss or addition of nutrients, or by intracellular processes, such as rescue of stalled ribosomes (26, 27, 30, 43, 45, 56).

Antimicrobial tolerance/persistence has been found in almost all bacteria, including human, animal, and plant pathogens (26, 27, 29–31, 36, 37); it is related to the ability of pathogens to withstand and perhaps subvert host defenses (40, 42, 57). The potential clinical relevance of antimicrobial tolerance/persistence in chronic and relapsing infections has been demonstrated for a wide range of bacterial infections (33–35, 40, 48, 57–65), and the fact that bacteria displaying such tolerance may undergo mutagenesis at a higher frequency than usual to generate genetically coded antimicrobial resistance (26, 31, 40, 57) has stimulated extensive research focused on discovering antimicrobials active against such bacteria (29, 30, 66).

## POTENTIAL MECHANISMS OF ANTIMICROBIAL TOLERANCE/PERSISTENCE IN B. BURGDORFERI

Tolerance to metabolic, chemical and physical challenges, including antimicrobials, is a crucial if not obligatory phenotype of B. burgdorferi necessary for completion of its enzootic cycle in mammalian and possibly avian reservoirs and in ticks. The organism can remain viable in mice and unfed ticks for years despite host immunological responses (2, 67–70), and potentially antimicrobial-tolerant VBNC B. burgdorferi may be generated in mice by defects in the expression of RpoS (71). Several pathways and genes possibly involved in generation of B. burgdorferi antimicrobial tolerance have been identified. These include the stringent response mediated by *rel* and *dksA* (42, 72–77), synthesis of the quorum sensing factor AI-2 mediated by *luxS* (78, 79), and modulation of the levels of ATP and protein aggregation indirectly mediated by the GTPase *cgtA* (*obgE*) (43, 75, 80). Other factors that may be involved in the generation of antimicrobial tolerance in B. burgdorferi include decreases in the rate of growth triggered by scarcity of nutrients (81, 82) perhaps at least in part due to host antiborrelial antibodies blocking nutrient and ion transport, much as occurs in other bacteria (83, 84). Diauxic shifts in metabolism produced by availability of different sugars in the tick could also play a role in this process (74, 75, 85, 86).

### The stringent response.

This evolutionarily conserved response, mediated by *rel* and *dksA*, is triggered by amino acid starvation and other environmental stresses and functions in B. burgdorferi to regulate growth and inhibit DNA replication, transcription, and translation (44, 45, 72–77) and presumably facilitates, together with other regulators, B. burgdorferi permanence in ticks and vertebrate host reservoirs (74, 75, 86–92), since the stringent response is known to mediate these properties in a wide range of other bacteria (26, 44, 45, 92). The oxidative stress regulon modulated by BosR might also be tasked with this role in B. burgdorferi, since BosR is also regulated by the stringent response (26, 93, 94). In other bacteria, antimicrobial tolerance is mediated by the SOS response (26), but because both the SOS response and the toxin-antitoxin system are absent in B. burgdorferi despite its having a protein containing a MazE-like antitoxin domain (71), the stringent response currently appears to be the only known pathway for persistence in this pathogen (95–97).

The B. burgdorferi stringent response can be induced by amino acid starvation, but its triggering by other metabolic stimuli has not been fully examined (98–100). The presence of DksA in B. burgdorferi suggests that the B. burgdorferi stringent response may be stimulated by changes in pH and pO_2_ that produce conformational shifts in this protein, as in other bacteria (76, 77, 101, 102). Rel-mediated (p)ppGpp synthesis and degradation in B. burgdorferi might also generate a bistable regulatory circuit similar to a toxin-antitoxin module (45, 85), which, with the help of DksA-mediated transcription and nucleoid proteins, could result in population heterogeneity to antimicrobial challenges via modulation of DNA supercoiling. B. burgdorferi tolerance to antimicrobials could be similarly generated by (p)ppGpp together with hibernating factors by inactivating ribosomes (45, 56).

When triggered by amino acid starvation, the B. burgdorferi stringent response upregulates expression of peptide transporters (OppA1, -2, -3, and -5) (71, 74, 75, 87); recovery following doxycycline exposure is accompanied by induction of the oligopeptide permease genes *oppD* and *oppF* (16). Since B. burgdorferi expresses OppA2 at high levels in mice and ticks, it is reasonable to infer that this expression is partially the result of an activated stringent response that can generate borrelial tolerance to antimicrobials and other damaging agents in both ticks and vertebrate hosts.

### Metabolic modulation.

In many bacterial species, generation of antimicrobial tolerance/persisters is associated with alterations in the biosynthetic or metabolic status of the cell (16, 26, 86, 103). In Escherichia coli and Staphylococcus aureus, lowering ATP levels results in increased numbers of antimicrobial-tolerant bacteria, probably due to changes in transcription of select genes and protein aggregation mediated by changes in concentrations of DnaK-ClpB and ObgE (43, 48, 75, 80). While synthesis of (p)ppGpp modifies the GTP/ATP ratio in B. burgdorferi by consumption of GTP and by inhibition of GTPases such as ObgE (CgtA) (43, 75, 80), there is currently no evidence that fluctuations in ATP levels play a role in generation of antimicrobial-tolerant cells in B. burgdorferi. Sequential shifts in utilization of carbon sources (diauxie) could play this role in B. burgdorferi as they do in E. coli, since B. burgdorferi sequentially utilizes glycerol and chitobiose in ticks and glucose in the mammalian host (74, 75, 85, 86). The B. burgdorferi stringent response triggered by shifts in amino acid and fatty acid metabolism occurring during growth in ticks and mammalian tissues could also generate bacteria tolerant to damaging agents in those environments (72–75).

### Quorum sensing.

Mediators of quorum sensing are involved in the generation of antimicrobial tolerance in Streptococcus mutans and Pseudomonas aeruginosa (104, 105). B. burgdorferi does not seem to have a classical quorum sensing mechanism, although its LuxS can synthesize 4,5-dihydroxi-2,3 pentanedione (AI-2), a mediator of quorum sensing in other bacteria (78, 79, 94, 106–108). While *luxS* is activated during B. burgdorferi transfer from ticks to mice and AI-2 modulates expression *in vitro* of many B. burgdorferi genes required for virulence (79, 107–109), B. burgdorferi preferentially produces AI-2 during the exponential rather than the stationary phase of growth, whereas most tolerant cells appear during the stationary phase, and none of the genes activated by AI-2 in B. burgdorferi are associated with generation of antimicrobial-tolerant cells in other bacteria. Although ablation of B. burgdorferi *luxS* hampered the organism’s ability to disseminate in mice after intradermal injection, this was not related to apparent inability to generate cells tolerant to harmful effectors. LuxS may, however, influence biofilm formation and susceptibility to doxycycline in other bacteria and thus might be relevant where borrelial concentrations are high, e.g., in erythema migrans, early organ dissemination, and feeding-nymph guts (106, 109).

### Global regulators.

As in other bacteria, interactions among many global regulators with the stringent response and with each other might result in antimicrobial tolerance/persisters in B. burgdorferi (71, 94, 110–112). For example, both RpoS and the stringent response are involved in the formation of B. burgdorferi round morphotypes that may be tolerant to antimicrobials (71, 75, 88, 89, 94), and RpoS deficiencies potentially generate VBNC spirochetes in mice (71). CsrA, another B. burgdorferi global regulator, might cooperate with the stringent response in generating antimicrobial tolerance/persisters in the course of its modulation of motility, biofilm formation, and glucose utilization (113–117). Similarly, the ability of c-di-GMP and the Hk1-Rrp1 axis to modulate carbon utilization, motility, and potentially biofilm formation could suggest their involvement in the generation of antimicrobial tolerance in B. burgdorferi (94, 118–124). The question also arises of whether BadR (required for mouse infection and able to modulate expression of the stringent response), RpoS, BosR, and chitobiose utilization (diauxic shift) could be involved in generation of tolerance to injurious factors, including antimicrobials (70, 94, 111, 125, 126).

### Other possible mechanisms.

Host defenses, such as the antibacterial neutrophil protein calprotectin, can inhibit B. burgdorferi growth and make it tolerant to penicillin (127, 128), while antibodies blocking its nutrient transport systems could trigger the stringent response and result in antimicrobial-tolerant cells (83, 84, 87, 129, 130). Exposure of *B. burgdorferi* to reactive nitrogen and oxygen species, and acidic and osmotic stresses during its transient intracellular location in mammalian macrophages (131, 132) or during its traversal of the tick larval and nymphal gut could similarly be responsible for development of B. burgdorferi tolerance to antimicrobials and alterations in cell morphology (68, 132–134). Fluctuation in antimicrobial concentrations during treatment also may increase the frequency of B. burgdorferi tolerance to them in the host (135).

B. burgdorferi is pleomorphic and can assume multiple morphotypes under different culture conditions (75, 88–91, 136–138). Transition to round forms can be mediated by modulation of the stringent response and RpoS under conditions of nutrient depletion, and some of these borrelial morphotypes have been shown to elicit distinct immune responses in infected animals and perhaps in patients with Lyme disease (139, 140). The relevance of these morphological variants to antimicrobial tolerance and pathogenesis of Lyme disease remains unclear (141, 142). A recently identified ribosome-dependent modulation of bacterial cell geometry in response to ambient nutrient conditions could provide a mechanistic link between these phenomena (143).

## B. BURGDORFERI ANTIMICROBIAL TOLERANCE/PERSISTENCE IN CULTURE AFTER ANTIMICROBIAL EXPOSURE

Early studies indicated heterogeneity in B. burgdorferi cultures regarding their susceptibility to antimicrobials (144, 145). Examination of the kinetics of B. burgdorferi killing in response to doxycycline and amoxicillin demonstrated clear heterogeneity of the different strains to antimicrobial challenge (146). Killing of B. burgdorferi by cefodizime, ceftriaxone, penicillin, vancomycin or erythromycin followed a biphasic curve, similar to cultures of other bacteria containing cells tolerant to antimicrobials (144, 145, 147). That human neutrophil calprotectin reduced killing of B. burgdorferi by penicillin and that a small number of B. burgdorferi organisms in infected macrophages could survive and be cultured suggest that B. burgdorferi could become tolerant to β-lactams *in vivo* as well as providing a mechanism by which B. burgdorferi could resist intracellular host defenses (131). The occasional intracellular location of B. burgdorferi could also provide a niche to escape antimicrobial activity without metabolic alterations (131). These results, though not extensively cited, indicate that B. burgdorferi, like other bacteria, exhibits mechanisms that allow it to tolerate the antimicrobial activity of drugs and host defenses.

More studies have confirmed the presence of antimicrobial-tolerant cells in B. burgdorferi cultures. This was first suggested by the increased tolerance of stationary-phase B. burgdorferi to doxycycline, amoxicillin, or nitrofurantoin and by alterations in spirochete morphology, including round bodies (90, 91). Cultures exposed to doxycycline, amoxicillin, or ceftriaxone displayed biphasic killing curves typical of cultures containing tolerant cells whose numbers increased during the stationary phase and whose tolerance to antimicrobials was not heritable (14, 15). Emergence of B. burgdorferi cells tolerant to doxycycline in stationary-phase cultures was stochastic and bacterial-density dependent (14, 15). Such putative antimicrobial-tolerant cells could be killed by daptomycin, carbomycin, cefoperazone, vancomycin, or clofazimine individually or by a combination of doxycycline, daptomycin, and cefoperazone (148, 149). It is not clear whether pulsed antimicrobial treatment is effective in decreasing the numbers of antimicrobial-tolerant B. burgdorferi in these cultures, as apparently different results have been obtained with pulses of doxycycline and ceftriaxone (14, 15, 150).

While antimicrobial-tolerant B. burgdorferi persisters share many similarities with antimicrobial tolerance in other bacteria, they exhibit some unique features, including an apparently higher frequency and possible continuing susceptibility in culture to β-lactam antimicrobials, such as azlocillin (15, 151). In addition, antimicrobial-tolerant B. burgdorferi cells can be reactivated by replete media without antimicrobials after a lag period of about 6 days (14). The mechanisms behind this reactivation are not known.

Antimicrobial-tolerant B. burgdorferi organisms differentially express many genes, including some specifying transporters, as well as ones involved in DNA repair and protein synthesis (16). This suggests that acquisition of the tolerant phenotype in B. burgdorferi is an active process dependent on both up- and downregulation of genes (16, 74, 75, 98). It should be mentioned that azlocillin, a potentially effective antimicrobial against antimicrobial-tolerant B. burgdorferi, interacts with both the ClpX protease and the penicillin-binding protein PBP3, two gene products whose transcription is modulated by the B. burgdorferi stringent response (75, 151).

## B. BURGDORFERI ANTIMICROBIAL TOLERANCE/PERSISTENCE IN ANIMAL MODELS

While it seems clear that B. burgdorferi antimicrobial tolerance can take the form of antimicrobial-tolerant persister cells *in vitro*, its ability to tolerate antimicrobials in animals and humans and the relevance of this ability to explain aspects of Lyme disease, including the evolution of antimicrobial-treated Lyme arthritis and PTLDS, have been highly contentious (152–155). While animal models vary in terms of their relevance for linking the phenomenon of antimicrobial tolerance to persistence in Lyme disease in humans, studies in multiple species have demonstrated posttreatment persistence of the spirochetes. (17–20, 156, 157).

A potential link between *in vitro* bacterial tolerance to antimicrobials and persistence *in vivo* is suggested by the observation that B. burgdorferi can be detected in mice and other animals after apparently adequate antimicrobial treatment (17, 18, 157, 158). These studies are summarized in Table 1. In 1994, Moody et al. showed the ineffectiveness of doxycycline treatment for clearing experimentally infected mice (156). Eight years later, Bockenstedt et al. used xenodiagnosis to test the viability of persisting spirochetes obtained after doxycycline treatment (17). A subsequent study by Bockenstedt et al. found probable persistence by culture of antimicrobial-tolerant borrelias in one of 12 infected immunodeficient MyD88 knockout mice treated with oral doxycycline, and B. burgdorferi 16S rRNA DNA could be detected in the ear skin of 5 of 12 of these mice and B. burgdorferi *ospA* in the joints of all 12 (19).

**TABLE 1:** Evidence for antimicrobial tolerance/persistence of B. burgdorferi in antimicrobial-treated mice

Antimicrobials	Treatment	Methodology	Summary	Reference
Penicillin G, amoxicillin-clavulanic acid, ceftriaxone, oxytetracycline, doxycycline, chloramphenicol, erythromycin, azithromycin	Various doses administered by gavage or subcutaneously1–4 times daily for 5 or 14 days (peak doxycycline serum levels comparatively higher than with human dose)	Treatment for 5 or 14 days at 7 days post-infection Infection status assessed by culture of ear biopsy specimens, spleen at 14, 30, and 90 days posttreatment Histopathology of joints and heart at 14, 30 and 90 days posttreatment	High-dose penicillin G, amoxicillin-clavulanic acid or ceftriaxone eliminated B. burgdorferi and disease from infected mice Oxytetracycline, doxycycline, chloramphenicol, erythromycin or azithromycin failed to eliminate B. burgdorferi from infected mice	156
Controls	Saline injections containing only antimicrobials (no *B. burgdorferi*) or saline injections containing only *B. burgdorferi* (no antimicrobials)			

Ceftriaxone	16 mg/kg administered intraperitoneally twice daily for 5 days and then once daily for 25 days	Treatment by injection or by gavage for 30 days at 1 mo postinfection Infection status assessed by xenodiagnosis, followed by PCR and IFA of ticks and culture and qPCR of mouse tissues at 3, 6, and 9 mo after last treatment dose	Up to 3 mo after treatment, spirochetes visualized by microscopy in xenodiagnostic ticks from 4/10 antibiotic-treated mice could not be transmitted from these ticks to naive mice and lacked plasmid-associated genes correlating with infectivity. By 6 mo after treatment, mice no longer positive by xenodiagnosis By 9 mo after treatment, low levels of spirochete DNA detected by qPCR in 2/4 ceftriaxone- and 4/5 doxycycline-treated mice	17
Doxycycline	50 mg/kg, by gavage twice daily for 30 days			
Controls	Saline injections			

Ceftriaxone	16 mg/kg administered intraperitoneally twice daily for 5 days and then once daily for 25 days	Treatment by injection at early (3 wks) or chronic (4 mo) stages of infection with antimicrobial or saline for 1 mo Infection status assessed by culture, PCR, xenodiagnosis, transplantation of allografts 1 and 3 mo after treatment Tissues examined for spirochetes 1 and 3 mo after treatment by immunohistochemistry	Antibiotic-treated mice culture negative, but tissues from 2/5 remained PCR positive for borrelial DNA, spirochetes in these mice could be visualized by immunohistochemistry in collagen-rich tissues Spirochetes acquired from mice by xenodiagnoses (as determined by PCR) and xenodiagnostic ticks from these cohorts transmitted spirochetes to naive SCID mice which became PCR-positive but remained culture negative	18
Controls	Saline injections			

Tigecycline	12.5 mg/kg or 50 mg/kg doses administered subcutaneously 1× daily for 10 days.	Treatment by antimicrobial or saline control at 1 wk (early dissemination), 3 wks (early stage of infection) or 4 mo (chronic stage of infection) after infection. Infection status assessed 3 mo after treatment by culture, qRT-PCR, and subcutaneous transplantation of joint and heart tissue into SCID mice	Tissues from all antimicrobial-treated mice culture negative, but some tissues from most mice treated with antibiotics *ospA*-positive by PCR Viability of nonculturable spirochetes in antimicrobial-treated mice confirmed by transplant of tissue allografts into SCID mice, with dissemination of spirochetal DNA to multiple recipient tissues, and by xenodiagnoses Tissue from heart base of antimicrobial-treated mice showed transcription of several B. burgdorferi genes by RT-PCR Infected SCID mice did not display any pathological lesions	158
Ceftriaxone	16 mg/kg administered intraperitoneally twice daily for 5 days and then once daily for 25 days			
Controls	Saline injections			

Ceftriaxone	16 mg/kg administered intraperitoneally twice daily for 5 days and then once daily for 25 days	Treatment by antimicrobial or saline 30 days after infection Infection status assessed at 2, 4, 8, and 12 mo after treatment by culture, qRT-PCR, xenodiagnosis and immunofluorescence on xenodiagnostic ticks	B. burgdorferi not cultured from tissues, but low copy no. of B. burgdorferi *flaB* DNA detected by PCR in tissues at 2, 4, and 8 mo after treatment, rate of PCR-positive tissues progressively declined over time Resurgence of spirochete *flaB* DNA in multiple tissues at 12 mo, with *flaB* DNA copy levels nearly equivalent to those found in control saline-treated mice RNA transcription of multiple B. burgdorferi genes detected in host tissues, *flaB* DNA detected in xenodiagnostic ticks Spirochetal forms visualized within ticks and mouse tissues by indirect immunofluorescence and immunohistochemistry	159
Controls	Saline injections			

Further studies in mice by Hodzic et al. compared the efficacy of ceftriaxone treatment when given in the early phase (3 weeks) or the chronic phase (4 months) of infection (18). B. burgdorferi cells were detected in mouse collagenous tissue by immunohistochemistry, xenodiagnoses, PCR, and fluorescence microscopy at both times (18). In mice treated with tigecycline or ceftriaxone at various times after being infected with B. burgdorferi, Barthold et al. later found that cardiac tissue from antimicrobial-treated mice was PCR positive for persistent spirochetes and RNA transcription of several B. burgdorferi genes (158). Spirochetal viability was confirmed by transplantation of tissue allografts from these treated mice into severe combined immune-deficient (SCID) mice and by xenodiagnosis, which included acquisition by ticks, transmission by ticks to SCID mice, and survival through molting into nymphs and then into adults. Antimicrobial-tolerant B. burgdorferi cells remaining in mouse tissues were thus transcriptionally active and viable despite their nonculturability.

Hodzic et al. (159) demonstrated low copy numbers of B. burgdorferi *flaB* DNA in tissues of infected mice at 2, 4, and 8 months after treatment, with the rate of PCR-positive tissues declining over time. Importantly, however, resurgence of spirochete *flaB* DNA was observed in multiple tissues at 12 months, with *flaB* DNA copy levels being nearly equivalent to those found in untreated mice. Despite the nonculturable state of regrown borrelias, RNA transcription of multiple B. burgdorferi genes in multiple tissues was present, B. burgdorferi *flaB* DNA was detected in xenodiagnostic ticks, and spirochetal forms could be visualized within ticks and mouse tissues by immunofluorescence and immunohistochemistry, respectively. These antimicrobial-tolerant B. burgdorferi cells could multiply from continuing foci of infection and invade tissues without histological evidence of inflammatory pathology yet with increased expression of host inflammatory cytokines (32, 159). These putative antimicrobial-tolerant spirochetes remained viable for up to 18 months following treatment yet stayed nonculturable (32). Recent experiments have further confirmed the failure of doxycycline, ceftriaxone, and vancomycin to eradicate B. burgdorferi in mice infected with stationary-phase B. burgdorferi (139).

There is additional support for biologically active but nonviable borrelias and borrelial antigens remaining in mouse tissues after antimicrobial treatment (17–19). Intravital microscopy of B. burgdorferi-infected wild-type and MyD88 immunodeficient mice treated with doxycycline or ceftriaxone showed amorphous structures containing B. burgdorferi antigens adjacent to dermal ear cartilage and in knee joint entheses for extended periods of time after treatment in the absence of infectious bacteria (19). While borrelial peptidoglycan is shed when the spirochetes divide, how long it may linger *in vivo* is uncertain, as the inflammatory exudate may contain lysozyme (23). Nevertheless, the presence of borrelial peptidoglycan in tissues has been shown to induce arthritis in rats (160) and can be found in the joints of untreated and treated patients with Lyme arthritis (23). There is thus evidence to support multiple mechanisms by which viable antimicrobial-tolerant spirochetes as well as nonviable spirochetes and spirochetal antigenic debris in the tissues are capable of causing disease in mice and possibly in humans.

Rhesus macaques have been used as models of Lyme disease because they display all the manifestations of B. burgdorferi infection of human patients, including erythema migrans, carditis, arthritis, and peripheral and central nervous system disease (161–163). They also exhibit the same stages of disease as human patients (early, early disseminated, and late) and the same variability in their antibody response to several B. burgdorferi antigens, including VlsE C6 fragment, OspC, and DbpA (155, 164). In one of the first studies of antimicrobial efficacy in primates, macaques were needle-inoculated with 10^8^ virulent B. burgdorferi B31, treated with doxycycline 4 months after inoculation, and assayed 3 months later (20). Xenodiagnoses were positive in two of three macaques, cultures were positive for B. burgdorferi RNA in all three, and, while spirochetes could not be regrown from any of these animals regardless of treatment, B. burgdorferi RNA as well as DNA could be detected in their tissues (20). In 12 macaques inoculated with B. burgdorferi JD1, treated with sequential regimens of ceftriaxone and doxycycline 27 weeks after inoculation, and examined postmortem 6 months later, one macaque was positive for B. burgdorferi DNA by PCR, three were positive for B. burgdorferi RNA by RT-PCR, and seven were positive for B. burgdorferi antigens by immunofluorescence (20). Three animals in this last group had moderate to severe inflammatory lesions in their tissues.

**FIG 1:**
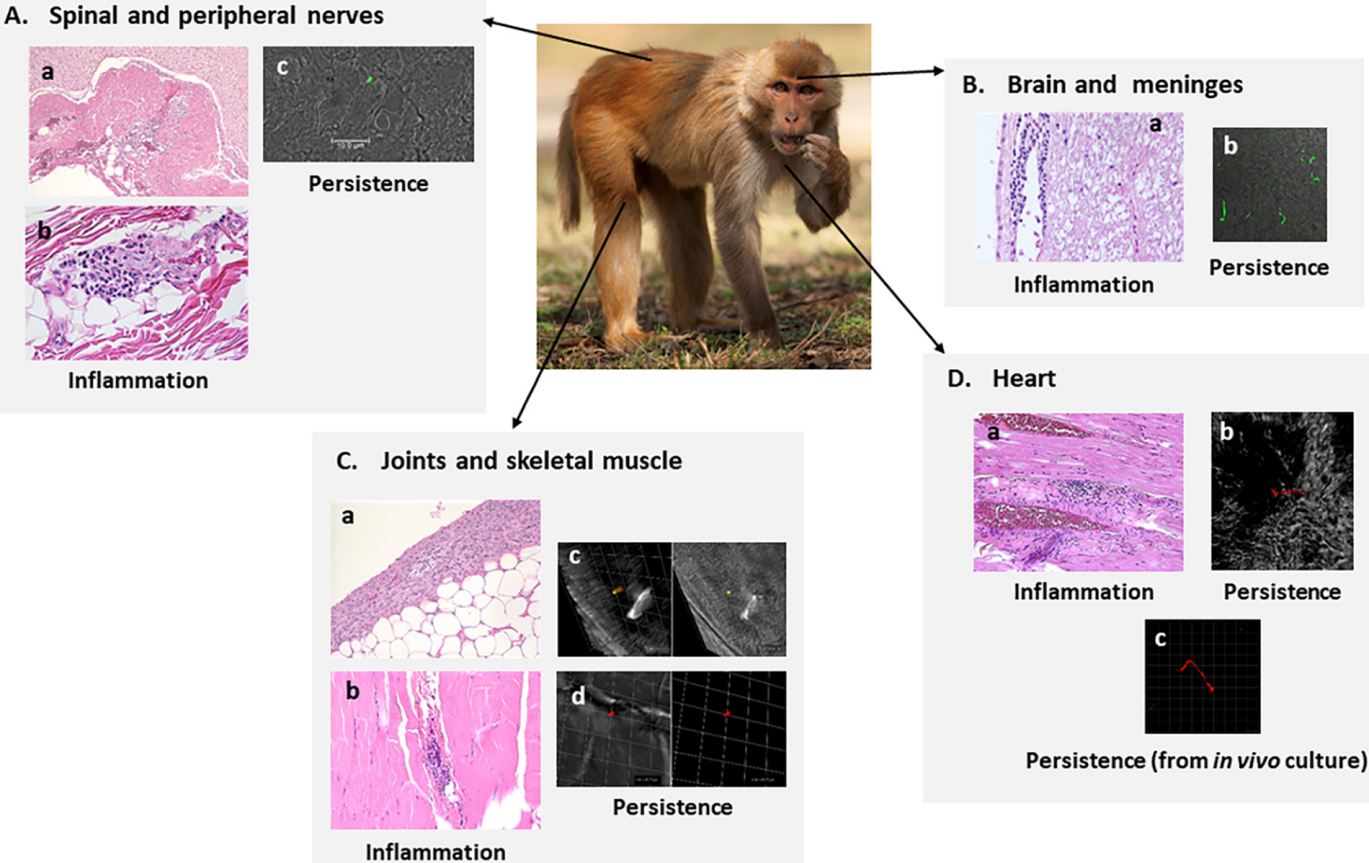
Inflammatory infiltrates and antimicrobial-tolerant persistent B. burgdorferi in tick-inoculated rhesus macaques 8 to 9 months after treatment with oral doxycycline (5 mg/kg of body weight, twice a day for 28 days) (12 to 13 months after inoculation) (163). (A) Spinal cord and peripheral nerves. (a) Mild inflammation surrounding a cervical spinal nerve. (b) Minimal to mild mononuclear inflammation in tibial nerve. Inflammation tended to be distributed perivascularly in perineural fibrous connective tissue. (c) Section of a spirochete in the spinal cord immunostained with rabbit polyclonal *B. burgdorferi*-specific antibody (164). (B) Brain and meninges. (a) Mononuclear perivascular cuffing in a focal area of the brain adjacent to the fourth ventricle of the medulla. (b) Multiple spirochetes in the cerebral parenchyma immunostained with rabbit polyclonal *B. burgdorferi*-specific antibody (164). (C) Joints and skeletal muscle. (a) Mild synovial hyperplasia with piling up of the synovial epithelium and minimal concurrent inflammation. (b) Minimal to mild mononuclear cell infiltration in skeletal muscle interstitium. (c and d) Three-dimensional reconstruction of an immunostained section of skeletal muscle to show the cross-section of a persistent spirochete identified by dual staining with rabbit polyclonal and mouse monoclonal anti-B. burgdorferi OspA antibodies. (D) Heart. (a) Localized interstitial mononuclear cell foci adjacent to a coronary blood vessel. (b) A persistent spirochete within the myocardium identified by IFA with mouse monoclonal anti-B. burgdorferi OspA antibody. (c) Persistent *B. burgdorferi* spirochetes from macaque heart tissue cultured in an *in vivo* culture system identified by IFA with a mixture of mouse monoclonal anti-B. burgdorferi OspA and anti-B. burgdorferi OspC antibodies (164). Samples of these cultures were positive for *ospA* and *oppA-2* transcripts identified by quantitative RT-PCR (data not shown) (163).

**FIG 2:**
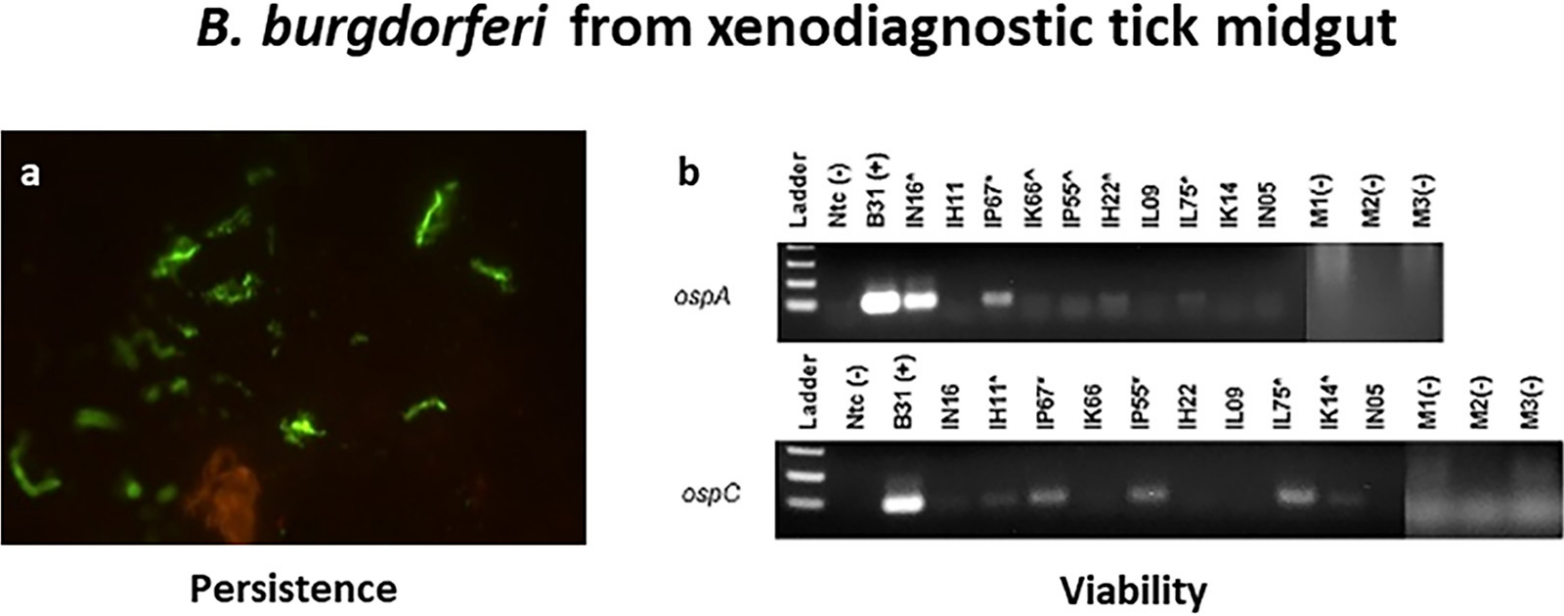
B. burgdorferi in xenodiagnostic tick midgut contents (163). (a) Antimicrobial-tolerant persistent spirochetes identified by IFA with mouse monoclonal anti-OspA antibody in ticks fed on treated rhesus macaques approximately 7 months postinoculation. (b) Viability of these spirochetes confirmed by RT-PCR for B. burgdorferi *ospA* and *ospC*. *, clear positive; ^, potential positive. M1, M2, and M3 represent cohort-matched controls derived from feeding ticks on clean mice.

The presence of borrelias after antimicrobial treatment was also seen 4 months after infection in five monkeys infected with B. burgdorferi B31.5A19 by nymphal ticks and treated for 28 days (Fig. 1) (163). In addition to the presence of potential antimicrobial-tolerant spirochetes in these animals (determined by xenodiagnoses with nymphal ticks at 3 months and 7 to 8 months after treatment), spirochetes were demonstrable by immunofluorescence (164). Necropsy and histological analysis of these five infected and treated monkeys demonstrated foci of moderate inflammation in many organ and tissue targets of disseminated B. burgdorferi infection (164). A few of these tissues contained occasional borrelias detected by immunofluorescence. Importantly, multiple spirochetes were identified within the cerebral parenchyma of two doxycycline-treated macaques. In three animals, RT-PCR showed persistent spirochetal RNA, indicating biosynthetic activity (Fig. 2), but these B. burgdorferi organisms were not able to productively infect immunodeficient CB17 SCID mice (164). Biosynthetically active B. burgdorferi organisms were cultured from heart tissue samples from two of five treated monkeys using a technique in which tissue isolates were incubated in dialysis bags within the rat peritoneal space.

Arguments against the existence of antimicrobial-tolerant B. burgdorferi in infected mice and monkeys have included claims that residual cells were not really tolerant to antimicrobials because the animals had been insufficiently treated with such agents (165). However, pharmacokinetic analysis of doxycycline in macaques confirmed that the doses were adequate (166). While it has been asserted that the inability to culture these putative antimicrobial-tolerant B. burgdorferi cells argues against their existence (167), apparently VBNC forms have recently been shown to occur in B. burgdorferi, a well-known and frequent phenomenon in other bacteria where the existence of antimicrobial-tolerant cells is uncontroversial (36–38, 41–43, 71).

The failure to culture putative antimicrobial-tolerant B. burgdorferi from mouse tissues and xenodiagnostic tick midguts and the low infectivity of such cells could perhaps be due to generation of auxotrophic mutants or mutants less tolerant to harmful immunological factors in animals and ticks, since stress mechanisms involved in generating antimicrobial tolerance in bacteria are also known to mediate increases in mutation rates (26, 29, 30, 40, 58). A role for such auxotrophic mutants in the failure to culture these B. burgdorferi antimicrobial-tolerant cells is consistent with rescue of some of them from rhesus monkey heart tissue by passage through rat peritoneal incubation chambers (163). That B. burgdorferi mutants potentially deficient in RpoS and nutritional polypeptide transport OppA5 can generate VBNC bacteria in mouse tissues is consistent with this speculation (71). The apparent failure to culture antimicrobial-tolerant B. burgdorferi could also depend on the need for enzymatic resuscitation factors acting upon the peptidoglycan of the bacterial cell membrane, a situation known to occur in Mycobacterium tuberculosis cells potentially tolerant to harmful factors (168, 169).

Long-term detection of borrelial DNA in the tissues of infected animals after antimicrobial treatment has been ascribed to the long-term chemical stability of DNA in the tissues in the absence of viable organisms (19, 152, 170). There is the possibility that nucleic acid fragments could remain detectable in nonviable organisms if they were sequestered in some manner in the tissues. This seems unlikely, since multiple other studies have shown that injected purified DNA (including borrelial DNA) is rapidly cleared from animal and human tissues (158, 171–180), presumably by tissue DNases (171–180). This in turn suggests that borrelial DNA detected in the tissues derives from contemporaneous, initially antimicrobial-tolerant and metabolically active bacterial cells rather than from detritus of long-dead cells (20, 32, 155, 159, 163).

Recent reports of the association of borrelial mRNA with persistent borrelial DNA in animal tissues is consistent with the viability of these borrelias (20, 32, 159, 163). While the inability of B. burgdorferi to produce toxins has been put forward as an explanation for the minimal pathological alterations found in animal tissues infected with persistent bacteria (153), B. burgdorferi does produce immunogenic extracellular proteases (e.g., the serine protease HtrA) that degrade fibronectin and extracellular matrix proteoglycans, including decorin (181–183). Both Htr and fibronectin can stimulate *in vitro* production of chemokines and proinflammatory cytokines, and they could, together with host matrix metalloproteases induced by scarce B. burgdorferi cells in tissues, play a proinflammatory role in this situation (181–183). Metabolically active, antimicrobial-tolerant B. burgdorferi could similarly recruit plasminogen, which subsequently contributes to extracellular matrix (ECM) degradation/inflammation (184).

## B. BURGDORFERI ANTIMICROBIAL TOLERANCE/PERSISTENCE IN PATIENTS

There is general agreement that B. burgdorferi can persist in untreated patients with Lyme disease for months and disseminate from its point of entry in the skin to generate late complications, such as arthritis and neuroborreliosis (2, 5, 8, 10). In contrast, persistence of B. burgdorferi after suitable antimicrobial treatment is highly contested and is the basis of heated controversies among the lay and scientific communities (5, 8, 10, 12).

B. burgdorferi has frequently been reported to remain in patient tissues after effective antimicrobial treatment, where it can be detected by culture (185–187), microscopy (188, 189), PCR (189, 190), immunoassay (189, 191), or xenodiagnoses (21, 192). Detection of B. burgdorferi peptidoglycan in synovial fluids of patients with Lyme arthritis despite the presence of lysozyme might also be indicative of currently viable or recently metabolically active bacteria (23). In one patient with PTLDS, the presence of B. burgdorferi DNA was demonstrated by xenodiagnoses on two occasions 8 months apart (21). Spirochetes could not be cultured from the ticks, and the ticks were not able to transmit spirochetes to SCID mice on either occasion. However, in light of the evidence reviewed above, it is difficult to envision survival of B. burgdorferi DNA for over a year in this patient in the absence of viable B. burgdorferi organisms or undetected reinfection (21, 172, 175, 177, 178, 192). In another group of patients (193), PCR/electrospray-mass spectrometry detected B. burgdorferi DNA 21 days after antimicrobial therapy in one patient. Treatment triggered a shift in the number of multiple coinfecting B. burgdorferi cells, suggesting that these infecting organisms had a different tolerance for doxycycline.

With regard to the delayed clearance of symptoms after antimicrobial treatment as a clinical manifestation of B. burgdorferi persistence resulting from antimicrobial tolerance, 10% of patients with Lyme arthritis who continued to have symptoms after 30 days of oral antimicrobials cleared their symptoms after a subsequent 28-day treatment with intravenous antimicrobials (24, 194, 195). Significantly, more children than adults with Lyme arthritis showed unresolved symptoms after antimicrobial treatment. Twenty-nine percent of children with Lyme arthritis remained symptomatic after antimicrobial treatment (112 of 383), and a second course of antimicrobials was able to eradicate symptoms in only 62% of these children (69 of 112) (24, 196).

To better understand these responses, Bouquet et al. (197) and Petzke et al. (198) compared longitudinal transcriptional analyses of peripheral blood mononuclear cells from patients with treated Lyme disease and controls with publicly available transcriptomic data from patients with other bacterial diseases and influenza. Both groups observed a distinct transcriptional signature in Lyme disease patients 3 to 4 weeks after treatment that differed from that seen in diseases caused by E. coli, S. aureus, S. pneumoniae, and influenza virus which returned to baseline by 6 months after treatment regardless of persistent symptomatology. In the 29 Lyme disease patients studied by Bouquet et al. (15 with resolved disease, 13 with persistent symptomatology, and one lost to follow-up), the Lyme disease signature had normalized with no significant differential gene expression patterns between the patients with resolved disease and those without, although pathways common to other chronic immune-mediated diseases remained perturbed in all. In the 11 patients studied by Petzke et al. (10 with resolved disease and 1 with persistent symptomatology), the disease signature also returned to baseline levels by 6 months after treatment, again regardless of persistent symptomatology. While these studies are consistent with the absence of B. burgdorferi toxin-induced pathology, they offer little insight into the possible antimicrobial tolerance and persistence of the spirochete or its remnants in treated patients and the modifications of these responses by antimicrobials (19, 21–23).

These findings raise the possibility that B. burgdorferi populations in infected patients may be heterogeneous with respect to their tolerance to antimicrobials/persistence and that, at least in some human hosts, certain variants might be able to persist after what otherwise would be adequate antimicrobial treatment (193, 197–200).

## CONCLUSIONS

The relevance of antimicrobial tolerance-mediated persistence in B. burgdorferi-infected patients remains contentious among both scientists in the field and the wider public (5–13). Evidence for persisting antimicrobial-tolerant borrelias from Lyme disease patients, while not definitive, is consistent with observations in model animal systems and a wide range of *in vitro* studies. The efficacy of repeated and extended antimicrobial treatment to cure some cases of Lyme arthritis and to improve manifestations of PTLDS in some patients is also consistent with borrelial persistence mediated by antimicrobial tolerance (12, 194–196). Further study will be needed to establish this connection as well as the effectiveness of such prolonged treatment in a subset of PTLDS patients with potentially antimicrobial-tolerant B. burgdorferi. The possible causes of PTLDS are multiple, and antimicrobial-tolerant borrelial persistence cannot be ruled out as a factor. Application of recent results of *in vitro* and animal studies to clinical research can be expected to clarify the role of bacterial persistence and antimicrobial tolerance in PTLDS.

B. burgdorferi is endowed with several genetic and metabolic mechanisms that in other bacteria are responsible for generation of antimicrobial tolerance. Apart from *in vitro* and animal experiments, their relevance to the presence of antimicrobial-tolerant B. burgdorferi in humans remains to be experimentally established. There is, however, strong experimental evidence from *in vitro* studies (14–16), animal models (17–20, 32, 155–159), and patients (21, 185–187) that B. burgdorferi can become tolerant to antimicrobials and remain in host tissues for extended periods of time in dynamic equilibrium with the host immune response (70, 155–159). This is underlined by the presence of B. burgdorferi DNA and RNA in xenodiagnostic ticks fed on animals and patients with potential antimicrobial-tolerant B. burgdorferi organisms, since tick midgut contents and salivary glands are likely to contain tissue nucleases able to clear naked DNA and RNA not associated with viable organisms (172, 175, 201). While the continuing presence of borrelial DNA in humans, animals, and ticks has been compared to that of bacterial DNA found in valves of patients with treated bacterial endocarditis years after treatment (202–204), this comparison is at best inexact, since valvular tissues are potentially immunologically privileged sites where access to antimicrobials and host endonucleases is limited and where DNA (even if generated by unculturable organisms) could be protected from degradation.

One promising approach is to generate mutants of B. burgdorferi genes potentially involved in antimicrobial tolerance (e.g., *rel*, *dksA*, and *rpoS*) in isogenic strains of *B. burgdorferi*. The ability of these strains to generate antimicrobial-tolerant persisters in vitro and in animals can be compared with that of wild-type strains and their epistatic interactions, regulatory hierarchies, and potential epigenetic markers assessed (39, 205, 206). Potential epigenetic modifications, such as DNA methylation of isogenic antimicrobial-tolerant borrelias, could similarly be assessed by nanopore-based DNA sequencing (39), while genome-wide mutagenesis and genome editing could permit identification of new genes and functions involved in antimicrobial tolerance-mediated persistence *in vitro* and *in vivo* in animals (206, 207). The recent rescue of potential antimicrobial-tolerant B. burgdorferi persisters by culture of heart tissues from chronically infected macaques can be expected to facilitate metagenomic and metatranscriptomic analysis and identification of the genetic elements underlying their inability to be readily cultured as well as those involved in persistence in mammals (156, 208, 209). It can also be expected to enable identification of therapeutic modalities capable of blocking functions needed for antimicrobial-mediated spirochetal persistence, tolerance, and revival and thus forestall development of PTLDS in some patients (9, 15, 16, 58, 66, 70).
